# Association between received treatment elements and satisfaction with care for patients with knee osteoarthritis seen in general practice in Denmark

**DOI:** 10.1080/02813432.2021.1922835

**Published:** 2021-07-05

**Authors:** Linda Baumbach, Donna Ankerst, Ewa M. Roos, Lillemor A. Nyberg, Elizabeth Cottrell, Jesper Lykkegaard

**Affiliations:** a Research Unit for Musculoskeletal Function and Physiotherapy, Department of Sports Science and Clinical Biomechanics, University of Southern Denmark, Odense, Denmark; b Department of Health Economics and Health Services Research, University Medical Center Hamburg-Eppendorf, Hamburg, Germany; c Department of Mathematics and School of Life Sciences, Technical University of Munich, Munich, Germany; d Faculty of Medicine and Health, Department of Medical Sciences, Örebro University, Örebro, Sweden; e Primary Care Centre Versus Arthritis, Keele University, Keele, UK; f Department of Public Health, Research Unit for General Practice, University of Southern Denmark, Odense, Denmark

**Keywords:** Osteoarthritis, patient satisfaction, therapeutics, association, general practitioners

## Abstract

**Objective:**

While education, exercise, and weight reduction when indicated, are recommended first-line treatments for knee osteoarthritis patients, they remain poorly implemented in favour of pain killer treatment, imaging and referral to surgery. A reason could be that patients are more satisfied with receiving these adjunctive treatment elements. This study aimed to investigate the associations between the received elements of care and the patient’s satisfaction with the care for knee osteoarthritis in general practice.

**Design:**

Cross-sectional study.

**Setting:**

A Danish general practice.

**Subjects:**

All consecutive patients ≥30 years of age who consulted the general practitioner (GP) with chronic knee complaints during 18 months and who replied to a mailed questionnaire (*n* = 136).

**Main outcome measures:**

The questionnaire addressed patient’s knee-related quality of life, and overall satisfaction with care, as well as reception of seven types of information, which are known quality indicators. Information on reception of adjunctive treatment elements was obtained from electronic medical records.

**Results:**

Patient satisfaction (versus neutrality/dissatisfaction) was positively associated with reception of information on: physical activity and exercise (relative risks [RR] 1.38, 95% bootstrap percentile interval [BPI] 1.02–4.33), and the relationship between weight and osteoarthritis (1.38, 1.01–4.41). No significant associations were found for the five remaining types of information and all the adjunctive treatment elements.

**Conclusion:**

Providing information as education to patients with knee osteoarthritis as part of the treatment is positively associated with satisfaction with care.

**KEY POINTS**
General practitioners worry about the doctor–patient relationship when addressing recommended lifestyle changes. However, this study revealed:
•Patients in general practice with knee osteoarthritis are satisfied with care after having received information on lifestyle changes, such as exercise and the relationship between weight and osteoarthritis.
•Patient satisfaction was not associated with the reception of adjunctive treatment elements for osteoarthritis.

## Introduction

Clinical guidelines recommend patient education, exercise, and weight reduction (if overweight) as first-line treatments for patients with knee osteoarthritis (OA) [1–5]. These treatments have been shown to reduce pain, increase physical function, and improve knee-related quality of life [1,2,6–8]. If adherence to first-line treatments is limited or does not lead to the desired improvement, adjunctive treatments, such as pain-relieving drugs or referral for surgical evaluation, should be considered [1,3–5]. However, the implementation of recommended step-wise treatment approaches leaves room for improvement, as first-line treatments often are not applied before the introduction of adjunctive treatment elements [9–14].

Among several reasons for the lack of implementation of first-line treatments for knee OA [15–19] is a patient preference for adjunctive treatment elements [20]. General practitioners (GPs) worry that encouraging lifestyle changes may negatively affect the doctor–patient relationship [16,21,22]. Further, GPs perceive that patients prefer other options than the first-line treatments [16]. However, patient satisfaction with knee OA-related care has not been comprehensively researched [22]. Such information would provide an indication that first-line treatments are acceptable to patients with knee OA.

This paper investigates the association between the received elements of care and the patient’s satisfaction with the care for knee osteoarthritis in general practice in Denmark.

## Materials and methods

### Study design

The study was cross-sectional and reported according to STROBE guidelines [23].

### Setting and data source

More than 98% of all Danish citizens are listed with a GP and most services are publicly funded. This study was conducted in one clinic with six GPs located in a town in southern Denmark with 2693 citizens as of 2019. The clinic was selected because it uses electronic medical records (EMR) allowing free text search, and codes all diagnoses according to the International Classification of Primary Care (ICPC-2-R). In the clinic, an average consultation proceeded from 10 to 15 min.

Prior to the study, the GPs and staff had a three-hour meeting with researchers to discuss and update clinical guidelines for the management of knee OA as part of a quality improvement project. Study data were obtained from the clinic’s EMR and a patient questionnaire, which was distributed after the meeting. The governmental region of Southern Denmark provided aggregated demographic summaries on the clinic’s listed patients compared to other clinics in the region.

### Participants

Participants were identified through EMR search, with an inclusion criterion of at least 30 years of age with a first or follow-up knee OA consultation between 1. September 2017 and 28. February 2019. They were identified by searching the EMR. Specifically, patients with an ICPC-2-R code of L90 (knee OA), L91 (OA) in combination with the word ‘knee’ mentioned in free text, or a recurrent L15 (knee complaint) with no adequate trauma or other explanation, were considered to have knee OA and thus included. The search was repeated every 6 months (1 March 2018, 1 September 2018, and 1 March 2019). After each search, included patients were mailed a questionnaire, with a reminder after 4 weeks to non-responders (Supplementary figure). Only the first response was included among patients who filled out the questionnaire more than once.

### Outcome variable

The primary outcome was patient satisfaction with knee-related care in response to the specific question in the mailed questionnaire: ’How satisfied are you with the treatments you received at the GP clinic concerning your knee problems?’, with answer options ‘very satisfied’, ‘satisfied’, ‘neither nor’, ‘unsatisfied’ or ‘very unsatisfied’. The outcome was dichotomized into ‘satisfied’ comprising the first two categories versus ‘unsatisfied or neutral’ comprising the last three categories.

### Independent variables of interest

The independent variables of interest compromised reception of 13 treatment elements, seven concerning the reception of patient information as education and six adjunctive treatment elements.

Information on patient education was obtained via questions from the OsteoArthritis Quality Indicator questionnaire (OA-QI), which has been validated for patients with knee, hip, or hand OA. [24]. Utilized questions of the OA-QI were: (1) ‘Have you been given information about osteoarthritis?’, (2) ‘Have you been given information about different treatment options?’, (3) ‘Have you been given any advice on how you might help yourself to manage or deal with your osteoarthritis?’, (4) ‘Have you been given information or advice about physical activity and exercise to help you with your joint pain?’, (5) ’Have you been given information on the relation between weight and osteoarthritis?’, (6) ‘Have you discussed and agreed with your GP when you will have a review of your joint pain and treatment?’ and (7) ‘Have you been advised to lose weight?’. Answers options were ‘yes’, ‘no’, and ‘I do not remember’ for the first six questions, and ‘yes’, ‘no’, and ‘I am not overweight’ with no further information for the last question. The answers were dichotomized into ‘yes’ versus ‘no or I do not remember’. Patients who answered they were not overweight to question seven were not included in the analysis group for that question.

Information on adjunctive treatment elements was obtained from past EMR records up to 6 months and included prescription of pain killers and referrals to a physiotherapist, orthopaedist, rheumatologist, X-ray, and MRI, each recorded as ‘received’ or ‘not received’. A referral was considered received if the EMR either included a referral note or a feedback note from the related specialist.

### Confounding variables

The following five confounders were considered: (1) age, (2) sex, (3) number of EMR recorded knee-related contacts to the GP and all other therapists, including orthopaedics, during the last half-year, (4) knee-related quality of life evaluated by the subscale of the Knee injury and Osteoarthritis Outcome Score (KOOS) [25], and (5) presence of a total knee replacement.

### Statistical methods

Descriptions of patient characteristics and confounding variables of satisfied versus neutral/unsatisfied patients were presented by means, standard deviations, percentages as appropriate, with tests of significance performed using two sample *t*-tests and *z*-tests for comparing means and proportions, respectively.

Univariate and multivariate logistic regressions were conducted to estimate the relative risk (RR) of being satisfied versus neutral/unsatisfied related to the reception of each treatment element. To ensure logistic regression estimation stability, only treatment elements with at least 10 events per outcome (received treatment element and satisfied, received treatment element and neutral/unsatisfied, not received treatment element and satisfied, not received treatment element and neutral/unsatisfied) were considered as recommended for good statistical practice [26]. For the treatment elements with insufficient sample size to be considered for logistic regression, Fisher’s test was used for determining associations between categorical independent variables and the dependent variable.

For all treatment elements with sufficient power, unadjusted and adjusted RR’s were reported alongside 95% bootstrap percentile intervals (BPI). Identified statistically significant treatment elements were further investigated regarding their independence with the Chi-square or Fisher’s test as appropriated and displayed in a Venn diagram.

Finally, sensitivity analyses excluding patients with neutral satisfaction were performed for the treatment elements significantly associated with satisfaction in the adjusted primary analyses.

All statistical tests were performed at the 0.05 level of significance using R statistical software (Version 1.1.463 2009-2018).

### Ethics

All included patients provided informed written consent for use of their data for the research project. The scientific ethical committee of the Region of Southern Denmark declared that no approval was needed. The study was approved by the legal services Research and Innovation Organisation from the University of Southern Denmark (case 10.267).

## Results

By 1 January 2019, the clinic with six GPs had 6240 listed patients. Of those 4174 were 30 years or older and 51% were female. The age distribution of the listed patients matched that of the whole region of Southern Denmark.

During the 18-month inclusion period, in total 242 (6%) of all listed patients, the ones aged ≥30 years had a registered encounter with the clinic due to knee OA and received an invitation to participate in the study. Of those, 136 (56%) answered the questionnaire and gave informed consent; 26 were invited and answered more than once, with only their first response used for analysis. Five patients were excluded due to missing information on the outcome satisfaction with knee-related care. Of the 131 patients with outcome data, 7 (5%) were very unsatisfied, 7 (5%) were unsatisfied, 40 (31%) were neutral, 59 (45%) were satisfied and 18 (14%) were very satisfied with their received knee-related care. This led to 77 (59%) satisfied and 54 (41%) unsatisfied or neutral included patients, with characteristics in Table 1.

**Table 1.** Characteristics of the satisfied and unsatisfied patients.

	All patients *n* = 131	Satisfied patients *n* = 77	Unsatisfied patients *n* = 54	*p*-Value (95%CI)
Age, mean (95% CI)	63 (40–85)	63 (43–85)	64 (40 − 85)	0.649 (−5.6 to 3.5)
Sex, *n* (%)				
Female	70 (53)	46 (60)	24 (44)	0.121 (−0.03 to 0.34)
Male	61 (47)	31 (40)	30 (56)	
Number of knee related GP contacts during half a year (95% CI)	2.1 (1.0–6.8)	2.3 (1.0–7.0)	1.9 (1.0–6.0)	0.105 (−0.01 to 1.00)
Knee related quality of life score (0 − 100, worst to best), mean (95% CI)	48 (13 − 87)	48 (6.3–83)	46 (13–94)	0.555 (−5.29 to 9.80)
	Missing 8	Missing 7	Missing 1	
Presence of at least one knee protheses, *n* (%)				
Yes	14 (12)	10 (15)	4 (9)	
No	100 (88)	57 (85)	43 (91)	0.46 (−0.07 to 0.20)
Missing	17	10	7	

CI: confidence interval; SD: Standard deviation.

Based on insufficient sample sizes of less than 10 events per outcome, the following four treatment elements were excluded from the logistic regressions: receipt of advice to lose weight, receipt of the information on when the next review should happen, referral to a rheumatological specialist, and referral to MRI (Table 2).

**Table 2.** Summary statistics and univariate relative risks for association of treatment elements with patient satisfaction.

Treatment elements	All patients	Satisfied patients	Unsatisfied patients	*p*-Value and unadjusted/adjusted^b^ Relative risk (95% BPI)
Obtained from the quality indicator questionnaire –				
Received information concerning…				
…osteoarthritis, *n* (%)^2+^				0.189
Yes	36 (28)	25 (69)	11 (31)	1.27 (0.92–1.70)
No	93 (72)	51 (55)	42 (45)	1.17 (1.00–2.79)^b^
…treatment options, *n* (%)^2+^				0.012*
Yes	56 (43)	40 (71)	16 (29)	1.49 (1.11–2.00)*
No	73 (57)	35 (48)	38 (52)	1.21 (1.00–2.98)
…managing osteoarthritis, *n* (%)^4+^				0.334
Yes	39 (31)	26 (66)	13 (34)	1.20 (0.89–1.59)
No	88 (69)	49 (56)	39 (44)	1.15 (0.93–2.22)^b^
…physical activity and exercise, *n* (%)^1+^				0.024*
Yes	74 (57)	50 (67)	24 (33)	1.46 (1.08–2.11)*
No	56 (43)	26 (46)	30 (54)	1.38 (1.02–4.33)^b^^,^*
…reducing weight (in case of overweight), *n* (%)^4+^				0.025*
Yes	25 (20)	20 (80)	5 (20)^a^	
No	54 (43)	28 (52)	26 (48)	
Not overweight	48 (38)	27 (56)	21 (44)	
…the relation between the weight and OA, *n* (%)^6+^				0.013*
Yes	45 (36)	33 (73)	12 (27)	1.50 (1.13–2.03)*
No	80 (64)	39 (48)	41 (52)	1.38 (1.01–4.41)^b^^,^*
…when the next review of your joint should happen, *n* (%)^3+^				0.582
Yes	15 (12)	10 (67)	5 (33)^a^	
No	113 (88)	64 (56)	49 (44)	
Obtained from the EMR regarding prescriptions and referrals for/to…				
… pain killers, including opioids, *n* (%)^2+^				0.410
Yes	81 (63)	45 (55)	36 (45)	0.86 (0.65–1.15)
No	48 (37)	31 (65)	17 (35)	0.93 (0.55–1.22)^b^
… physical therapy, *n* (%)				0.611
Yes	56 (43)	31 (55)	25 (45)	0.90 (0.66–1.23)
No	75 (57)	46 (61)	29 (39)	0.95 (0.58–1.23)^b^
… orthopaedic specialist, *n* (%)				0.966
Yes	33 (25)	20 (61)	13 (39)	1.04 (0.73–1.43)
No	98 (75)	57 (58)	41 (42)	1.02 (0.69–1.74)^b^
… rheumatological specialist, *n* (%)^2+^				1
Yes	5 (4)	3 (60)^a^	2 (40)^a^	
No	124 (96)	73 (59)	51 (41)	
… X-ray, *n* (%)^1+^				0.876
Yes	39 (30)	24 (62)	15 (38)	1.06 (0.77–1.40)
No	91 (70)	53 (58)	38 (42)	0.94 (0.51–1.20)^b^
… MRI, *n* (%)^1+^				0.164
Yes	2 (2)	0 (0)^a^	2 (100)^a^	
No	128 (98)	77 (60)	51 (40)	

BPI: Bootstrapped percentile interval; OA: osteoarthritis.

^n+^Number of missing values; ^a^to few cases per outcome for performing logistic regression, ^b^adjusted for age, sex, number of knee related contacts, knee related quality of life, and presence of a total knee replacement.

*Statistically significance *p* < 0.05.

None of the informational treatment elements was received by more than 57% of the included patients. The most often received treatment element was a prescription of pain medication. None of the adjunctive treatment elements was significantly associated with satisfaction (Table 2). Of the first-line treatments, the reception of two individual treatment elements was positively associated with patient satisfaction, namely information concerning (1) physical activity and exercise (RR 1.38, 95% BPI 1.02 to 4.33), and (2) the relationship between weight and osteoarthritis (RR 1.38, 95% BPI 1.01 to 4.41).

In sensitivity analyses excluding patients who reported ‘neutral’ satisfaction, neither of the two statistically significant types of information as treatment elements remained significant.

**Figure 1.**
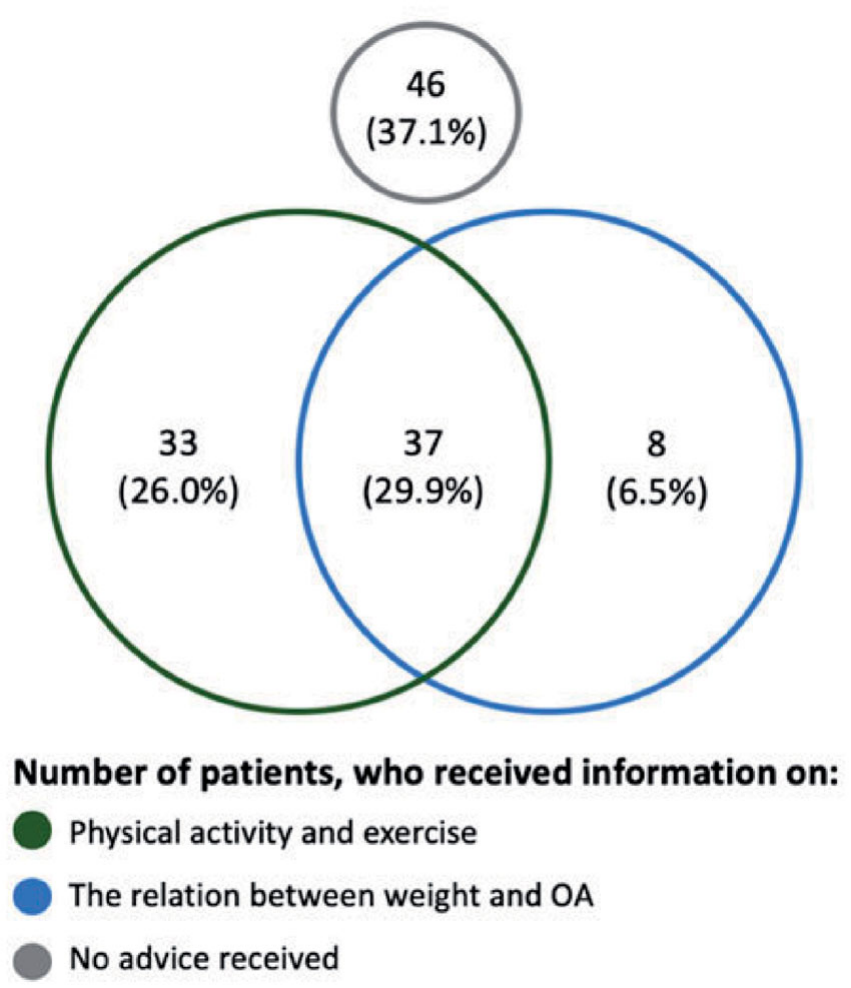
Venn diagram showing dependence of the statistically significant information as educational treatment elements (*n* = 131 of which seven are missing due to missing values).

The two statistically significant types of information as educational treatment elements were correlated, meaning that if a patient received one type of information, the chance was higher than the patient also received the other type (*p* < 0.001) (Figure 1).

## Discussion

### Key results

Knee-OA patients’ satisfaction with care was positively associated with reception of the following two pieces of information as first-line educational treatment elements: information concerning physical activity and exercise, and information on the relationship between weight and osteoarthritis.

### Interpretation

In line with the observations of earlier studies, this study indicates an underutilization of the recommended first-line treatments for patients with knee OA in general practice [13,27]. However, findings from this study contradict GP beliefs about patients generally getting irritated when advised on lifestyle [16,22]. GP beliefs were similarly demonstrated as incorrect in a study of patients presenting in general practice with a high risk of cardiovascular disease in Australia [28,29]. While the patients reported a willingness to change their lifestyle, GPs did not assume so and therefore withheld providing lifestyle advice [28]. Our findings indicated that this could very well be the same for GPs treating patients with knee OA in Denmark.

Patients included in this study had different durations of knee complaints before reporting to the study, some short and some long, and only few were first-time cases. Patients with longer durations of complaints were likely to have consulted other healthcare providers with the same complaints, which may have influenced their answers about satisfaction with care. However, in Denmark, the GP is the gatekeeper of treatment for chronic conditions. Treatment by other healthcare providers generally requires a referral from the GP. The GP decides whether to manage the patients themselves or to have management implemented by other healthcare professionals. Either way, the GP is responsible. Thus, the treatment and information provided to the patient through the GP referral should be regarded as provided by the GP. The phrasing of the satisfaction question in this study may have confused some patients as to whether or not they were asked only about satisfaction with actions happening in the GP clinic. However, such confusion is not likely to have biased the overall result and does not compromise the conclusion that overall satisfaction was positively associated with providing information as an educational treatment element.

The loss of significance in the sensitivity analyses, when those patients reporting neutral satisfaction were excluded, should be interpreted with caution as this finding may well be due to lack of power as the sample size reduced from 131 to 71 patients. The Venn diagram in Figure 1 shows that not all patients who received information on physical activity and exercise also stated that they received information on the relationship between weight and OA. Some patients could have only remembered the perceived most important pieces of information [30]. Further research is needed to investigate this hypothesis.

### Strengths

The study results are likely to represent real-world data as all consecutive patients were identified by a thorough review of the EMR, which also ensured high completeness of data. Free text records were reviewed to establish the OA diagnoses.

By teaching the guidelines to the GPs and staff before the study, it was more likely that a standardised high quality of information could be given to the patients.

The study population is representative of the Danish provincial population, as indicated by the age and gender composition. Furthermore, no other GP clinics are located in the study area, increasing the study’s completeness. Several potential confounders were respected.

### Limitations

In general, our results should be interpreted with caution. The cross-sectional design cannot address whether the association between treatment element and satisfaction represents a causal relation. The retrospective questionnaire is prone to recall bias, which may be unbalanced between satisfied and unsatisfied patients, as unsatisfied patients may be less likely to remember that they received a treatment element. There is a risk of selection bias and reduced generalisability, as only 56% of the eligible patients replied and agreed to participate in the study. Also, the small study sample prohibited the evaluation of treatment elements with less than 10 cases per outcome as required for stability of statistical estimation and inference. The small number of GPs increases the risk that satisfaction is linked to the GP rather than treatment. However, many patients encountered more than one of the GPs, which made it virtually impossible to connect patients with a specific GP.

The primary study outcome ‘satisfaction with received knee-related care’ was assessed with a single item Likert scale. Based on the specific question used here it remains uncertain whether a patient-reported satisfaction with the delivery or the outcome of the received care. More differentiated tools could have enabled discrimination between different aspects of satisfaction [15].

The definition of knee OA might further introduce a limitation, as we included patients with chronic knee pain from the age of 30, but did not require an explicit OA diagnosis in the EMR. However, already at an age of 30 years half of the athletes with a knee injury such as an ACL rupture have knee OA [31,32]. Nonetheless, we acknowledge that this inclusion might lead to a limitation especially when it comes to evaluating the reception of information on OA specific elements, such as how to manage OA, as clinicians might not assume this to be relevant. The option of reporting to not be overweight on the question regarding received information on weight reduction without further explanation or definition of overweight is an additional minor limitation.

Some patients probably had a referral prior to the study period and were therefore likely to be classified as not having received a referral. We reduced this potential misclassification by additionally classifying patients as referred if a feedback note was received in the EMR during the study period. Finally, the prescriptions for pain medications could have been made due to other conditions than knee problems. Thus, findings regarding pain killer prescriptions need to be interpreted with caution. Our study did not address what the patients expected and treatment asked for, nor if it was provided, when presenting with knee complaints at the GP clinic.

### Generalisability

Results of the study should be generalized with caution, especially if the underlying health care system differs from the Danish, or the patients are likely to differ from provincial Danes with regard to health literacy and expectations regarding healthcare.

## Conclusion

Providing information on physical activity and exercise and the relationship between weight and knee osteoarthritis as elements of first-line treatment is associated with increased satisfaction with care in patients with knee osteoarthritis.

## Supplementary Material

Supplemental Material
