# Supplementary figures and images for: Association between received treatment elements and satisfaction with care for patients with knee osteoarthritis seen in general practice in Denmark
Source: Scand J Prim Health Care. 2021 Jul 5;39(2):257–64. doi: 10.1080/02813432.2021.1922835 (PMC8293968; doi:10.1080/02813432.2021.1922835)

# Supplementary figure 1


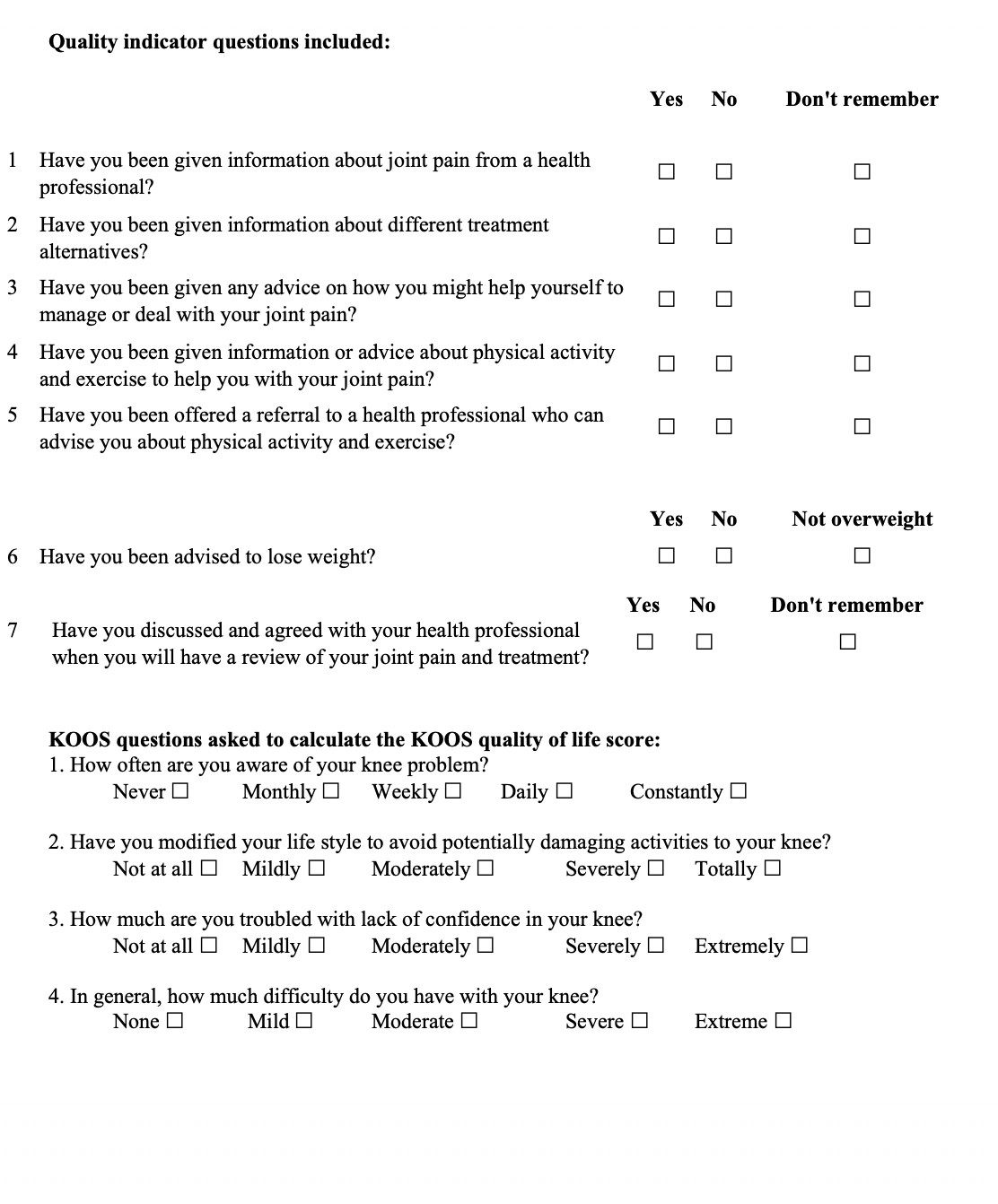

Supplement: Supplemental Material [file IPRI_A_1922835_SM6543.docx]
